# Excessive Smartphone Use is Associated with Depression, Anxiety, Stress, and Sleep Quality of Australian Adults

**DOI:** 10.1007/s10916-023-02005-3

**Published:** 2023-10-20

**Authors:** Asaduzzaman Khan, Geoffrey McLeod, Tarissa Hidajat, Elizabeth J Edwards

**Affiliations:** 1 School of Health and Rehabilitation Sciences, The University of Queensland, St Lucia, QLD 4072 Australia; 2 School of Education, The University of Queensland, St Lucia, QLD 4072 Australia

**Keywords:** Depression, Anxiety, Stress, Sleep Quality, Smartphone use

## Abstract

Problematic smartphone use has been associated with poorer mental health in different population groups; however, little is known about how levels of smartphone use were associated with mental health outcomes of adults in Australia. Using data from a cross-sectional survey among Australian adults aged 18–59 years (n = 655, Mean = 24.55 [SD = 5.59] years; 66% female), the current study aimed to examine association between problematic smartphone use and different psychological outcomes. Participants completed measures of problematic smartphone use with Mobile Phone Problem Use Scale (MPPUS), mental health outcomes with Depression, Anxiety, and Stress Scale (DASS-21), and Pittsburgh Sleep Quality Index (PSQI), in addition to some socio-demographics. Smartphone use was categorised into three groups: low-moderate, moderate-high, and high-severe. A total of 160 adults (24.4%) reported high-severe smartphone use. Multivariable linear regression analyses showed that smartphone use was inversely associated with psychological outcomes in a dose-dependent manner with high-severe smartphone uses having the most adverse effects. Compared to low-moderate use, average depression score was 3.5 points higher for moderate-high smartphone use (β = 3.51, 95% CI: 1.63–5.40) and 6.9 points higher for high-severe smartphone use (β = 6.91, 95% CI: 4.74–9.07). Similarly, average stress score was 3.4 points higher for moderate-high smartphone use (β = 3.40, 95% CI: 1.75–5.06) and 7.0 points higher for high-severe smartphone use (β = 7.02, 95% CI: 5.11–8.93). Similar association estimates were found for anxiety and sleep quality. Reducing smartphone use has the potential to optimise depression, anxiety, stress, and sleep quality; however, longitudinal research is warranted to establish directionality of the association.

**Supplementary Information:**

The online version contains supplementary material available at 10.1007/s10916-023-02005-3.

## Introduction

Smartphone use has increased exponentially in the past years. Statista [1] reported that more than six billion people across the world have a smartphone subscription with some countries have higher levels of smartphone use. In Australia, about 88% of its population own a smartphone, suggesting a high prevalence of smartphone usage [2]. The use of smartphones has brought many benefits including facilitating communication, easing access to information, managing time and work, enhancing student learning, as well as for social and entertainment purposes to name a few [3–6]. Regardless, smartphone use has also been linked to various negative consequences, such as traffic accidents, lowered physical activities, and lowered academic performance among other problems [6–9]. High smartphone use has also been linked to problematic smartphone behaviours [10]. For instance, frequent repetitions of smartphone behaviour, such as constant phone checking, can form problematic smartphone habits [11]. These smartphone habits may then lead to excessive and addictive smartphone usages, which include involuntary behaviours triggered by external or internal cues—for instance, automatic and repetitive unlocking of smartphones to check for notifications, even during inappropriate times such as when socializing with others [11, 12].

Problematic smartphone use (PSU) has been defined as the excessive use of smartphones that shows similar characteristics to substance abuse disorder, which results in the dysfunction and impairment of daily living [13]. Many studies have used the 27-item Mobile Phone Problem Use Scale (MPPUS) [4, 14–16] to assess PSU. In a large sample of Australian adults aged 18–83 years who completed the MPPUS, 19.6% were found to be at risk or problem users of their smartphone [3]. Participants reported ‘*feeling more problems when occupied on their mobile phones’* and ‘*more lost when their mobile phones are not in their possession’* [3].

Other work has attempted to find an explanation for the formation of PSU. It has been suggested that poor self-regulation may be associated with PSU, due to low inhibition, high impulsivity, and poor decision-making skills [11, 17–19]. It has also been proposed that individuals experiencing loneliness or social isolation may be vulnerable to PSU, as smartphone use allows individuals to connect with others, and provides an immediate alleviation of feelings of loneliness [18, 20]. Available research indicates that PSU is associated with psychological distress [13, 20]—which has been defined as a reflection of mental health problems, and includes symptoms of depression, anxiety, and stress [21]. One study using randomized online surveys reported that 74% of adults displaying PSU showed symptoms of mental health problems—namely poor self-control, anxiety, depression, and dysfunctional impulsivities—where indicators of these mental health problems were also able to predict the existence of PSU [19]. More specifically, PSU has been associated with depression [20, 22–24], anxiety [13, 20, 22, 24], stress [22, 24–26], and sleep problems or lowered sleep quality [23, 24, 27]. Given that individuals with mental health problems are prone to behavioural avoidance and social isolation, it is possible they may also be less motivated to use their smartphones, that is, limited checking of emails and less engagement with social media [28, 29]. Taken together, there is some evidence to suggest that higher smartphone use is related to poorer mental health; however, there are inconsistencies in the literature.

Some recent studies have investigated PSU in Australian adults [10, 30, 31]. With regards to the link between PSU and psychological outcomes, studies have shown that problematic smartphone behaviours were associated with feelings of anxiousness when being apart from their smartphones [10, 31] and that PSU was negatively related to psychological well-being more broadly—which has been defined as positive affect and effective functioning, and includes autonomy, environmental mastery, personal growth, purpose in life, self-acceptance, and social relations [30, 32, 33]. Previous studies conducted in Asian, European, and North American contexts had investigated the relationships between PSU and depression [20, 22–24], anxiety [13, 20, 22, 24], stress [22, 24–26], as well as difficulties with sleep [23, 24, 27]. However, to date no studies have examined the relationships between PSU and the emotional states of depression, anxiety, stress, and sleep quality in Australian adults and importantly explored the dose-dependence of such relationships. Hence, the current study aimed to examine the association of PSU with psychological symptoms —namely depression, anxiety, and stress—and sleep quality. Drawing on previous literature we hypothesise that higher use of smartphones would be adversely associated with depression, anxiety, stress, and sleep quality.

## Materials and Methods

### Procedures

A cross-sectional survey was conducted among Australian adults, from 20 January to 23 March 2021, using the Qualtrics online platform. Recruitment advertisements were posted on the University’s virtual learning environment (Blackboard) and social media (e.g., Facebook, Twitter). Participants were recruited using a variety of social media and forum sites, as well as face-to-face interactions (i.e., approaching and asking). Recruitment and data collected in this case are representative of a snowball technique, where participants were asked to share and forward the survey to anyone who they thought might be interested in completing it. Inclusion criteria include adults aged 18 years or above and have spoken English for work or study for at least five years. Age below 18 years was considered as an exclusion criterion for the online survey. The survey included some demographic questions (e.g., age, sex, education, employment) in addition to measures of smartphone use, mental health indicators including depression, anxiety, stress, and sleep. It took approximately 15-minutes to complete the online survey. The survey was approved by The University of Queensland Human Research Ethics Committee (#2,020,003,023, 18 January 2021).

### Participants

Of the 817 participants who provided informed consent and attempted the online survey, a total of 655 completed the variables of interest, which constitute the analytical sample for the current analyses (response rate: 80.2%). The participants represent a diverse group of people with different academic and socio-economic backgrounds. A total of 163 (24.9%) participants reported their education as high school graduate or less, while 291 (44.4%) reported bachelor’s degree or above. A total of 212 (32.4%) participants reported working full time, and 111 (17.0%) reported working part-time. English was re[ported as first language by 572 (87.3%) of participants (Table 1).

**Table 1:** Description of study sample

Characteristics	n (%)
Average Age (SD) [*n* = 649]	24.55 (5.59)
Sex [n = 640]	
Male	220 (34.38)
Female	420 (65.63)
English as first language [*n* = 655]	
Yes	572 (87.33)
No	83 (12.67)
Education [*n* = 655]	
Up-to high school	163 (24.89)
Trade or vocational	201 (30.69)
Graduation or above	291 (44.43)
Employment [*n* = 655]	
Unemployed	134 (20.46)
Casual	198 (30.23)
Part-time	111 (16.95)
Full-time	212 (32.39)
Problem Smartphone Use (MPPUS) [*n* = 655]	
Low to moderate	194 (29.62)
Moderate to high	301 (45.95)
High to severe	160 (24.43)
Average sleep (hrs/d) at night (SD) [*n* = 652]	7.12 (1.22)
Average sleep quality index (SD) [*n* = 652]	7.51 (3.33)
Average depression score (SD) [*n* = 655]	14.46 (11.35)
Average anxiety score (SD) [*n* = 655]	9.47 (8.20)
Average stress score (SD) [*n* = 655]	16.20 (10.05)

SD: Standard deviation

Total may not be equal to 655 due to missing data

### Measures

Mobile Phone Problem Use Scale (MPPUS) [14] was used to assess PSU. Respondents rate how often, in general, statements related to their smartphone usage (e.g., *My friends don’t like it when my smartphone is switched off* and *There are times when I would rather use the smartphone than deal with other more pressing issues*), on a 10-point Likert scale (0 = *not true at all* − 10 = *extremely true*). Item scores were summed to generate a global score and higher scores represent higher PSU. In our study, the MPPUS items had high internal consistency (Cronbach’s α = 0.91). MPPUS global score was categorised into three groups: low-moderate (27–76), moderate-high (77–126), and high-severe (> 126) in order to assess dose-response [14, 34].

Depression, Anxiety, Stress Scale (DASS-21) [35] is a 21-item scale measuring depression, anxiety, and stress across respective 7-item subscales. The items are reported on a 4-point Likert scale (where 0 = *Did not apply to me at all*, 1 = *Applied to me to some degree or some of the time*, 2 = *Applied to me to a considerable degree or a good part of time*, and 3 = *Applied to me very much or most of the time*) indicating how much the statement applied to participants over the past week (e.g., *I couldn’t seem to experience any positive feeling at all* and *I was unable to become enthusiastic about anything*). Item scores on the subscales were doubled to gain equivalence to the 42-score DASS total. Higher scores on each subscale represent greater symptoms of depression, anxiety, or stress. In our study, the subscale items showed good internal consistency (Cronbach’s α = 0.92, 79, and 0.87, respectively for depression, anxiety, and stress subscales).

Pittsburgh Sleep Quality Index (PSQI) [36] assessed participants sleep quality and quantity over the last month. Responses are both open-ended and on a 4-point Likert scale, (where 1 = *not during the last month*, 2 = *less than once a week*, 3 = *once or twice a week*, 4 = *three or more times a week*) e.g., *During the past month, how often have you had trouble sleeping because you cannot get to sleep within 30 min*. 18 of the 19 items were scored and categorised into seven subscales relating to sleep: quality, latency, duration, efficiency, disturbances, medication, and daytime dysfunction. A global score was then calculated from these component scores, with higher scores representing poorer overall sleep. The present study used the global score to index sleep quality.

The survey included a number of demographic variables including age, sex, English as first language, education, work status, occupation, and duration of sleep.

### Data Analysis

Descriptive statistics were computed for the variables of interest; means and standard deviations (SD) for continuous variables and proportions for categorical variables. To examine association of PSU with depression, anxiety, stress, and sleep quality, we used a series of linear regression models, adjusted for a set of covariates. Ordinary least-squares linear regression modelling was implemented with robust standard errors. In addition to examining distribution of the outcome measures (i.e., dependent variables), multicollinearity was assessed prior to building the regression models. Occupation was considerably associated with education and work status, and as such, occupation was excluded from any further analyses to avoid any issue of collinearity. Assumptions of the linear regression model were examined before finalizing the parameter estimates. Outliers were determined by analysing studentized residuals; data points with absolute values of studentized residuals greater than two were excluded from the fitted model. Breusch–Pagan/Cook–Weisberg test was used to examine heteroskedasticity of the fitted models. Finally, we conducted sensitivity analyses using different categories of PSU using percentiles: occasional use (< 15th percentile), habitual use (15th to < 80th percentile), at risk (80th to < 95th percentile), and problematic use (> 95th percentile) [37] to examine whether different categorisations impacted the results. The analysis was conducted using Stata v17SE (StataCorp, USA). The association estimates are presented in the form of crude and adjusted regression coefficients (β) and their 95% confidence interval (CI).

## Results

Descriptive statistics of the analytical sample (*n* = 655) are presented in Table 1. The mean age of participants was 24.55 (*SD* = 5.59, range 18–59 years) years, 65.6% were females and 34.4% were males. A total of 160 adults (24.4%) reported high-severe smartphone use while 301 adults (46.0%) reported moderate-high use of smartphone.

**Fig. 1:**
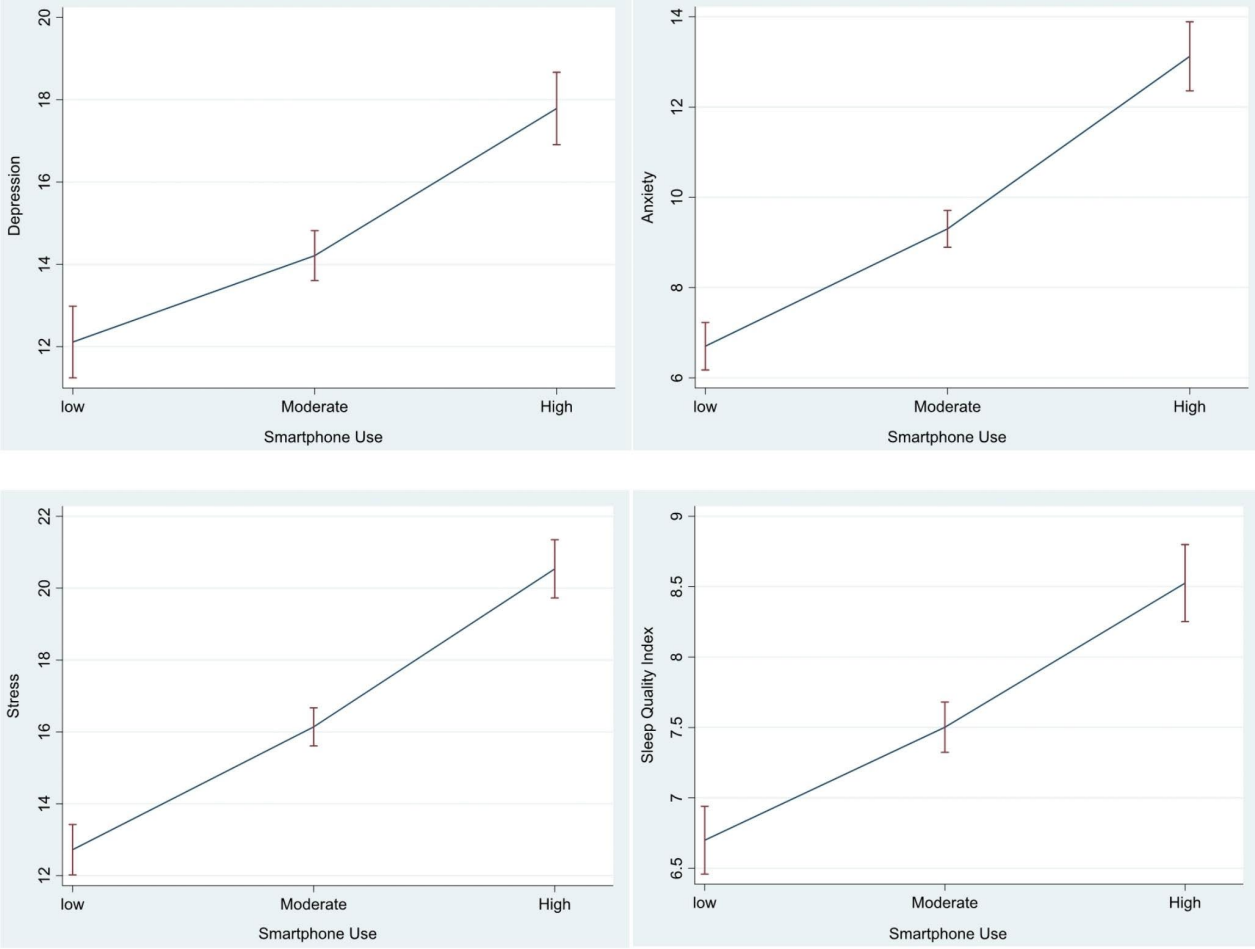
Average of psychological distress (DASS) score and sleep quality index at low, moderate, and high use of smartphone in study participants

**Fig. 2:**
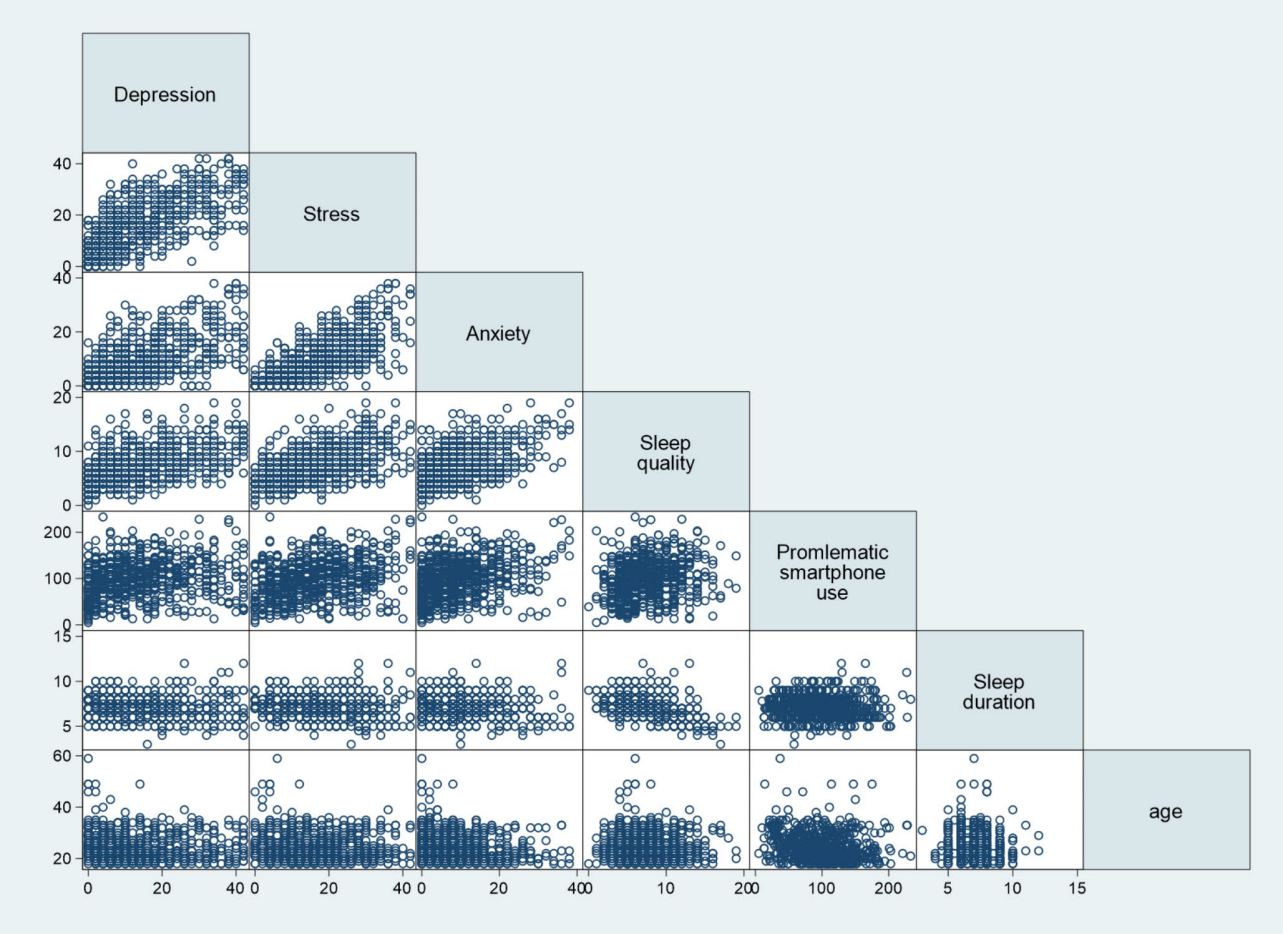
Scatterplots for all continuous variables

High use of smartphone was adversely associated with mental health outcomes (Fig. 1). For example, average depression score was 12.1 for low-moderate smartphone use, 14.2 for moderate-high use and 17.8 for high-severe use of smartphone. Similarly, average sleep quality was 6.7 for low-moderate smartphone use, 7.5 for moderate-high use and 8.5 for high-severe smartphone use. Figure 2 offers graphical representation of the relationships between all continuous variables through a series of scatterplots.

Multivariable linear regression analyses showed that smartphone use was inversely associated with psychological outcomes in a dose-dependent manner with high-severe smartphone use having the most adverse effects (Table 2). Compared to low-moderate use, average depression score was 3.5 points higher for moderate-high smartphone use (β_*adjusted*_ = 3.51, 95% CI: 1.63–5.40) and 6.9 points higher for high-severe smartphone use (β_*adjusted*_ = 6.91, 95% CI: 4.74–9.07). Similar adverse effects of smartphone were found for anxiety and stress in a dose-dependent manner. Additionally, average sleep quality was 0.72 points worse for moderate-high smartphone use (β_*adjusted*_ = 0.72, 95% CI: 0.27–1.17) and 1.4 points worse for high-severe smartphone use (β_*adjusted*_ = 1.40, 95% CI: 0.87–1.93).

**Table 2:** Regression coefficients of associations between depression, anxiety, stress, sleep quality and levels of smartphone use

Model: DV	Smartphone use	Crude Estimates	Adjusted Estimates^¥^
		Reg Coeff: β [95% CI]	Reg Coeff: β [95% CI]
1: Depression	[*Low-moderate* = Ref]		
	*Moderate-High*	3.62 [1.80–5.45]	3.51 [1.63–5.40]
	*High-Severe*	7.65 [5.55–9.75]	6.91 [4.74–9.07]
2: Anxiety	[*Low-moderate* = Ref]		
	*Moderate-High*	2.90 [1.73–4.08]	2.14 [0.93–3.34]
	*High-Severe*	5.74 [4.37–7.12]	4.65 [3.24–6.07]
3: Stress	[*Low-moderate* = Ref]		
	*Moderate-High*	4.46 [2.84–6.09]	3.40 [1.75–5.06]
	*High-Severe*	8.79 [6.90-10.69]	7.02 [5.11–8.93]
4: Sleep quality	[*Low-moderate* = Ref]		
	*Moderate-High*	0.97 [0.44–1.49]	0.72 [0.27–1.17]
	*High-Severe*	2.12 [1.51–2.73]	1.40 [0.87–1.93]

CI Confidence interval

DV dependent variable

^¥^Each of the linear regression models was adjusted for age, sex, education, employment, English as first language, and sleep duration

Depression, anxiety, and stress scores ranged from 0 to 42, while sleep quality index ranged from 0 to 21

Sensitivity analyses with different categories of smartphone use produced similar results without meaningful changes (Table S1). Adverse associations of smartphone use with psychological outcomes were observed in a dose-dependent manner. For example, compared to occasional smartphone use, average anxiety score was 2.3 points higher for habitual use smartphone use (β_*adjusted*_ = 2.28, 95% CI: 0.78–3.78), 4.9 points higher for at risk smartphone use (β_*adjusted*_ = 4.91, 95% CI: 3.03–6.78) and 6.8 points higher for problematic smartphone use (β_*adjusted*_ = 6.77, 95% CI: 3.99–9.54).

## Discussion

The current study examined whether different levels of smartphone use were associated with depression, anxiety, stress, and sleep quality in Australian adults. The results supported our hypothesis that higher smartphone use was adversely associated with depression, anxiety, stress, and sleep quality in a dose-dependent manner, such that greater smartphone use resulted in more severe symptoms of depression, anxiety, stress, and poor sleeping quality. The findings in the current study were consistent with findings from previous work, where higher smartphone use was found to be associated with depression/anxiety and sleep quality in working adults and university students. Specifically, our results with Australian adults are conceptually similar to past studies conducted in other contexts, which found that PSU was linked to elevated depression [20, 22–24], anxiety [13, 20, 22, 24], stress [22, 24–26], and difficulties with sleep [23, 24, 27], and importantly in a dose-dependent manner [22, 23]. Our results also concur with studies examining the relationship between PSU and psychological well-being more broadly—such as affect, satisfaction with life, autonomy, and environmental mastery—in Australian adults [10, 30, 31].

Regarding the development of internet addiction in response to negative situations, Kardefelt-Winther [38] suggested a theory of compensatory internet use, where maladaptive use of internet or technology is motivated by the desire to escape negative affect—regardless of the accompanying consequences, such as in the lack of real-life social interactions. It has been suggested that a similar phenomenon may be present in PSU, where smartphones are used to cope with depression, anxiety, and stress, which may then result in PSU [39, 40]. For instance, people with depression may want to avoid face-to-face communications and rely on their smartphones to cope with their negative mood [40], or that people who are feeling stressed may use video games on their smartphones to cope with their stress [41]. Additionally, anxiety has often led to the development of problematic behaviours (i.e., rumination, social isolation, fear of missing out, and boredom), which may result in PSU through frequent repetition of smartphone behaviours [13]. Hence, there may be bidirectional relationships between PSU and depression/anxiety, where PSU leads to depression/anxiety or that such psychological symptoms leads to PSU [39, 40].

Alternatively, there may be a cyclical relationship between depression/anxiety and/or sleeping problems with PSU [39, 40]. It is plausible that smartphone usage may provide positive reinforcements to users by alleviating negative emotion [22]. However, when smartphone behaviours are not possible/available, individuals with PSU may end up experiencing negative moods as a result of their unfulfilled longings [22]. That is, users are compelled to constantly use smartphones in order to alleviate or prevent negative moods [22]. Furthermore, it is possible that sleep problems from PSU can lead to stress, depression, or anxiety [42], or that PSU can lead to depression/anxiety that result in sleeping difficulties [43, 44]. Therefore, a variety of mechanisms may underly the relationship between PSU and mental health outcomes, which deserves further research.

By utilizing a large and diverse sample of Australian adults and using comprehensive full-scale measures of depression, anxiety, stress (using validated DASS) and sleep quality (using validated PSQI), and multivariable modelling adjusted for a set of covariates, the current study provides a robust assessment of association of PSU with depression/anxiety/stress and sleep quality. The current study makes a unique contribution to the current literature in an Australian context, and provides practical implications, especially when smartphone use is increasing exponentially. The dose-dependent relationships between smartphone usage and depression/anxiety as well as sleep quality found in the current study implies that Australian adults should regulate their amount of smartphone usage in efforts to maintaining psychological well-being.

### Limitations

Several shortcomings of the current research need mention. First, the design was cross-sectional, which prevents any inference about causation. Future research warrants a longitudinal design to examine the directionality and interrelationships of these behaviours across time. Second, the current study was based on a non-representative sample, which precludes generalisation of study findings. Third, the current study used self-report measures, albeit valid and reliable psychometric scales. Further work using objective measures of smartphone use and an experimental manipulation of anxiety/stress (e.g., ego-threat instructions to induce situational stress) would be of interest. Fourth, the current study included only a few variables to adjust the relationship between PSU and psychological outcomes, when there may be other variables that could potentially mediate the relationships [22, 45]. For example, it is possible that using smartphones for work may lead to stress from the work and but not the smartphone exposure [45]. Moreover, the demand to stay awake and work using smartphones may disturb sleep regulation [46]. Fifth, the scales used in the study have high internal consistency; however, their validity was not examined in the current study. Sixth, the distribution of anxiety score was approximately normal; however, this is unlikely to bias the estimates from linear regression models due to the moderately large sample (n = 655) of the current study, as supported by the central limit theorem. Last, the current study did not differentiate the types of smartphone usage, such as for social or non-social uses, as well as context [10, 20]. For instance, non-social use of smartphones, such as for work, news consumption, or relaxation, has been related with anxiety and PSU [11, 28]. On the other hand, the social use of smartphones, such as in the use of social media, is associated with feelings of jealousy or unhealthy social comparison, leading to increased depressive and anxiety symptoms [39, 47]. Therefore, future studies might need to consider associated variables which might mediate the relationships being examined and also between different types of smartphone usage in PSU with psychological outcomes.

## Conclusion

The current study contributes to the limited understanding of the relationship between smartphone use and mental health outcomes, and demonstrated that high smartphone use, specifically PSU, was associated with depression/anxiety/stress and sleep quality in Australian adults. Specifically, a dose-dependent relationship was observed with higher smartphone use resulting in more severe symptoms of depression, anxiety, stress, and poorer sleep quality. The current findings underscore the need for Australian adults to regulate their smartphone usage and be aware of the potential detrimental side effects from using smartphones excessively, so that the risk of developing problematic smartphone usage can be minimised. The findings can also inform behaviour modification interventions targeting healthy use of smartphones to maintain their overall psychological well-being. Randomised controlled trials are warranted to examine the directionality and the potential mechanisms underlying the relationships between smartphone use and mental health outcomes, which in turn can provide guidance for reasonable smartphone use and inform strategies to educate Australians about how smartphone can be used to optimise benefits.

### Electronic Supplementary Material

Below is the link to the electronic supplementary material.

Supplementary Material 1
