# Supplementary material for: Excessive Smartphone Use is Associated with Depression, Anxiety, Stress, and Sleep Quality of Australian Adults
Source: J Med Syst. 2023 Oct 20;47(1):109. doi: 10.1007/s10916-023-02005-3 (PMC10587281; doi:10.1007/s10916-023-02005-3)
Supplement: Supplementary file 1 — Supplementary Material 1 [file 10916_2023_2005_MOESM1_ESM.docx]

**Table S1** *Sensitivity analysis of associations between depression, anxiety, stress, and sleep quality and levels of smartphone use*

| Model: DV | Smartphone use | Crude Estimates | Adjusted Estimates^¥^ |
| --- | --- | --- | --- |
|  |  | Reg Coeff: β [95% CI] | Reg Coeff: β [95% CI] |
| 1: Depression | [*Occasional* =Ref] |  |  |
|  | *Habitual* | 5.00 [2.75-7.25] | 4.44 [2.10-6.78] |
|  | *At risk* | 8.30 [5.40-11.10] | 7.72 [4.84-10.60] |
|  | *Problematic* | 12.06 [8.17-15.95] | 8.98 [4.85-13.10] |
|  |  |  |  |
| 2: Anxiety | [*Occasional* =Ref] |  |  |
|  | *Habitual* | 3.64 [2.20-5.08] | 2.28 [0.78-3.78] |
|  | *At risk* | 6.61 [4.79-8.44] | 4.91 [3.03-6.78] |
|  | *Problematic* | 9.03 [6.26-11.80] | 6.77 [3.99-9.54] |
|  |  |  |  |
| 3: Stress | [*Occasional* =Ref] |  |  |
|  | *Habitual* | 5.02 [2.99-7.05] | 3.53 [1.45-5.60] |
|  | *At risk* | 9.43 [6.88-11.98] | 7.52 [4.96-10.08] |
|  | *Problematic* | 15.31 [11.63-18.99] | 10.31 [6.68-13.94] |
|  |  |  |  |
| 4: Sleep quality | [*Occasional* =Ref] |  |  |
|  | *Habitual* | 1.19 [0.56-1.82] | 1.05 [0.49-1.61] |
|  | *At risk* | 2.18 [1.40-2.97] | 1.82 [1.12-2.52] |
|  | *Problematic* | 3.35 [2.22-4.49] | 2.21 [1.19-3.24] |

CI Confidence interval

DV dependent variable

^¥^Each of the linear regression models was adjusted for age, sex, education, employment, English as first language, and sleep at night.

Depression, anxiety, and stress scores ranged from 0 to 42, while sleep quality index ranged from 0 to 21.
